# Poly(1-Napthylamine) Nanoparticles as Potential Scaffold for Supercapacitor and Photocatalytic Applications

**DOI:** 10.3390/mi13091528

**Published:** 2022-09-16

**Authors:** Ahmad Umar, Sundararajan Ashok Kumar, Daniel Rani Rosaline, Hassan Algadi, Ahmed A. Ibrahim, Faheem Ahmed, Edson Luiz Foletto, Savariroyan Stephen Rajkumar Inbanathan

**Affiliations:** 1 Department of Chemistry, College of Science and Arts, and Promising Centre for Sensors and Electronic Devices (PCSED), Najran University, Najran 11001, Saudi Arabia; 2 Department of Materials Science and Engineering, The Ohio State University, Columbus, OH 43210, USA; 3 Post Graduate and Research, Department of Physics, The American College, Madurai 625002, Tamil Nadu, India; 4 Post Graduate and Research, Department of Chemistry, Lady Doak College, Madurai 625002, Tamil Nadu, India; 5 Department of Electrical Engineering, College of Engineering, Najran University, Najran 11001, Saudi Arabia; 6 Department of Physics, College of Science, King Faisal University, Al-Ahsa 31982, Saudi Arabia; 7 Graduate Program in Chemical Engineering, Federal University of Santa Maria, Santa Maria 97105-900, RS, Brazil

**Keywords:** Poly(1-naphthylamine), Nanoparticles, Supercapacitor, Photocatalysis

## Abstract

Herein, we explore the supercapacitor and photocatalytic applications of poly(1-naphthylamine) (PNA) nanoparticles. The PNA nanoparticles were synthesized by using polymerization of 1-naphthylamine and characterized with several techniques in order to understand the morphological, structural, optical and compositional properties. The structural and morphological properties confirmed the formation of crystalline nanoparticles of PNA. The Fourier-transform infrared (FTIR) spectrum revealed the successful polymerization of 1-naphthylamine monomer to PNA. The absorption peaks that appeared at 236 and 309 nm in the UV–Vis spectrum for PNA nanoparticles represented the π–π^*^ transition. The supercapacitor properties of the prepared PNA nanoparticles were evaluated with cyclic voltammetry (CV) and galvanostatic charge–discharge (GCD) methods at different scan rates and current densities, respectively. The effective series resistance was calculated using electrochemical impedance spectroscopy (EIS), resulting in a minimum resistance value of 1.5 Ω. The highest specific capacitance value of PNA was found to be 255 Fg^−1^. This electrode also exhibited excellent stability with >93% capacitance retention for 1000 cycles, as measured at 1A g^−1^. Further, the prepared PNA nanoparticles were used as an effective photocatalyst for the photocatalytic degradation of methylene blue (MB) dye, which exhibited ~61% degradation under UV light irradiation. The observed results revealed that PNA nanoparticles are not only a potential electrode material for supercapacitor applications but also an efficient photocatalyst for the photocatalytic degradation of hazardous and toxic organic dyes.

## 1. Introduction

Nanotechnology has become an extremely interesting field in the past few years due to the nanometer-sized particles and their unique physical, chemical, electronic and magnetic properties resulting from their large surface area [1,2]. Nanoparticles (NPs) have been employed in a wide variety of applications, such as in environmental science, as catalysts, as energy storage devices and in the medicinal field [3]. The use of electronic devices is currently increasing day by day. As a result, there has been a growing desire for energy storage to be included as a key component of future energy systems alongside renewable energy sources. This would expand the use of renewable energy resources among families and business, as well as facilitate the widespread replacement of cars with internal combustion engines with low- or zero-emission cars. Consequently, a wide range of energy storage devices in various configurations will be required. Various energy storage systems have been developed to date for a variety of uses. The charge storage capacity in these batteries is high, but they take longer to charge and discharge. Therefore, we need to move to a new type of energy storage device, called a supercapacitor [4,5], which has functionalities such as fast charging and discharging, high power density and a long cycle life. Supercapacitors are classified into two types, electrical double layer capacitors (EDLCs) and pseudocapacitors [6]. Carbon materials have been used for EDLC applications due to their high power density and high surface area. EDLC stores charge at the electrode/electrolyte interface of the material through the separation of charges, exhibiting non-faradaic reactions. Carbon-based materials have good cycle stability but a low specific capacitance value due to their low energy density [7,8]. To increase the energy density and specific capacitance values of supercapacitors, pseudocapacitor materials, such as transition metal oxides (TMOs) and conducting polymers (CPs), must be established [9]. Pseudocapacitors store charges on the surface of the electroactive material, which exhibits a faradaic reaction, resulting in high energy density and high specific capacitance [10]. However, the cyclic stability is low in the case of pseudocapacitors [11,12,13,14,15,16,17,18,19,20]. In order to overcome these drawbacks, researchers have developed CPs for supercapacitor applications. With unique capabilities that make them appropriate for possible applications, conducting polymers (CP) have received much attention in recent decades. CPs such as polyacetylenes (PAcs), polyanilines (PANIs), polypyrroles, polyfluorenes (PFs), poly(arylenevinylenes) (PArVs), poly(phenylene ethylene vinylenes) (PEVs), polythiophenes (PThs), poly(1-naphthylamine) (PNA) and poly(carbazole) (PCz) have been extensively explored due to their unique optoelectronic and thermal properties, as well as their good environmental stability, good conductivity, high surface area, redox potential and adjustable parameters [21,22,23,24,25,26,27]. Moreover, these polymers have low solubility in typical organic solvents. Compared to many aniline derivatives, PNA has a wide range of applications, such as gas sensing, photocatalysis and energy storage, due to its unique electrical characteristics [28,29,30,31,32,33,34]. Hence, researchers have turned their focus to polymerization of 1-naphthylamine (1-NPA) monomer with fused benzene linked to aniline. PNA has all of the benefits of a semiconductor, including simplicity of production, adjustable redox capabilities, pH-sensitive electrochromic features and facile doping characteristics [35,36,37,38,39]. Various difficulties have arisen as a result of expeditious increases in the population and industrial development across the world, including pollution, energy shortages and infectious illness. In recent years, the environment has been affected by water pollution. Waste from dye enterprises is detrimental to the surrounding environment’s living organisms and can lead to human body malfunctions in a direct or indirect manner [40]. The major problem is the removal of contaminants from water. In this context, photocatalysis is one promising method to remove contaminants from water [41,42].

Until now, no studies have been published on the use of pristine PNA as an electrode and photocatalyst. The present work aims to investigate two applications of PNA, as an electrode material for supercapacitor and as a photocatalyst, for the degradation of methylene blue (MB) in water under UV irradiation.

## 2. Materials and Methods

### 2.1. Materials

All the chemicals were procured from Sigma-Aldrich and used as received without any further purification. For the polymerization of 1-napthylamine, several chemicals, including 1-naphthylamine (NA), ammonium peroxydisulfate ((NH_4_)_2_S_2_O_8_) and hydrochloric acid (HCl), were utilized. Deionized water was used as a solvent throughout the experiment. To prepare the supercapacitor electrodes, polyvinylidene difluoride (PVDF, Solvay) binder and conducting carbon (Super-P, TIMCAL) in a weight ratio of 70:15:15, respectively, were used with an appropriate amount of N-methyl 2-pyrrolidone (NMP). Methylene blue (MB) dye was used for photocatalytic degradation.

### 2.2. Synthesis of Poly(1-Naphthylamine) Nanoparticles

A simple technique was used to synthesize poly(1-naphthylamine) (PNA) nanoparticles. In a typical reaction process, 1M of 1-naphthylamine (NA) was dissolved in 1M HCl (1.67 mL of HCl in 20 mL of DI water). With steady magnetic stirring, 1M of ammonium peroxydisulfate (APS) was gently added to the NA monomer (1:1). The color of the mixture changed to purple blue, suggesting that PNA was formed. The entire mixture was then agitated for three hours and stored at room temperature for 12 h. The mixture was then purified by washing it four times with DI water and ethanol. The mixture was then dried in a hot air oven at 60 °C for 12 h.

### 2.3. Characterizations of Poly(1-Naphthylamine) Nanoparticles

The synthesized PNA nanoparticles were examined with various techniques in order to determine the structural, morphological, compositional and optical properties. The structural properties of the prepared nanoparticles were examined with X-ray diffraction (XRD, Rigaku-Ultima III max, ) with Cu (λ = 1.54 Å) radiation at an angular step size of 0.01 degrees in the range of 10–80 degrees. The morphology of the synthesized PNA nanoparticles was observed using field emission scanning electron microscopy (FESEM; Carl Zeiss Ultra Plus, Rave Scientific, USA). The chemical compositions of the synthesized PNA nanoparticles were investigated with Fourier-transform infrared (FTIR; Jasco-4600, Tokio, Japan) spectroscopy in the wavelength range from 400 to 4000 cm^−1^ at room temperature. To determine the optical properties, the synthesized material was examined using UV–Vis absorption spectroscopy (Perkin Elmer).

### 2.4. Fabrication of Poly(1-Naphthylamine) Nanoparticle-Based Electrodes for Supercapacitor Applications

In order to examine the electrochemical performance of the poly(1-naphthylamine) nanoparticles, an electrode was fabricated using PNA as a working electrode. To fabricate the electrode for the electrochemical tests, a slurry was formed by combining active material (Poly(1-naphthylamine), polyvinylidene difluoride (PVDF) binder and conducting carbon in a weight ratio of 70:15:15 with a sufficient amount of N-methyl 2-pyrrolidone (NMP). The mixture was then agitated for 12 h to produce a uniform slurry, which was subsequently coated on a 1 × 1 cm^2^ area of a graphite sheet with a painting brush. The fabricated electrode was dried in a vacuum oven at 80 °C for 1 h before being utilized for electrochemical experiments. All of the electrochemical experiments (CV, GCD and EIS) were carried out in a three-electrode setup (Model: CHI6008e, USA), consisting of a working electrode (active material), a counter electrode (a platinum wire) and a reference electrode (Ag/AgCl), in a 2M KOH aqueous solution.

### 2.5. Photocatalytic Activity of Poly(1-Naphthylamine) Nanoparticles

To investigate the photodegradation of methylene blue (MB) dye using poly(1-naphthylamine) nanoparticles as a photocatalyst, a standard stock solution with a dye concentration of 5 mg L^−1^ was prepared, and 10.0 mg of poly(1-naphthylamine) nanoparticles was dispersed in 100 mL of the MB dye solution. To attain adsorption–desorption equilibrium, the sample was stirred for 30 min in a dark room. A photocatalytic experiment was conducted under UV light. To check the degradation of the targeted dye, 2 mL of the supernatant was collected from the reaction mixture after a regular interval of UV light exposure and centrifuged at 3000× *g* rpm for 5 min to separate the dye solution and the photocatalyst. The UV light was irradiated using a HPSLIV16254 photo-reactor equipped with an 11 W mercury UV lamp with a wavelength of 376 nm.

## 3. Results and Discussion

### 3.1. Characterizations and Properties of PNA Nanoparticles

#### 3.1.1. XRD Studies

**Figure 1:**
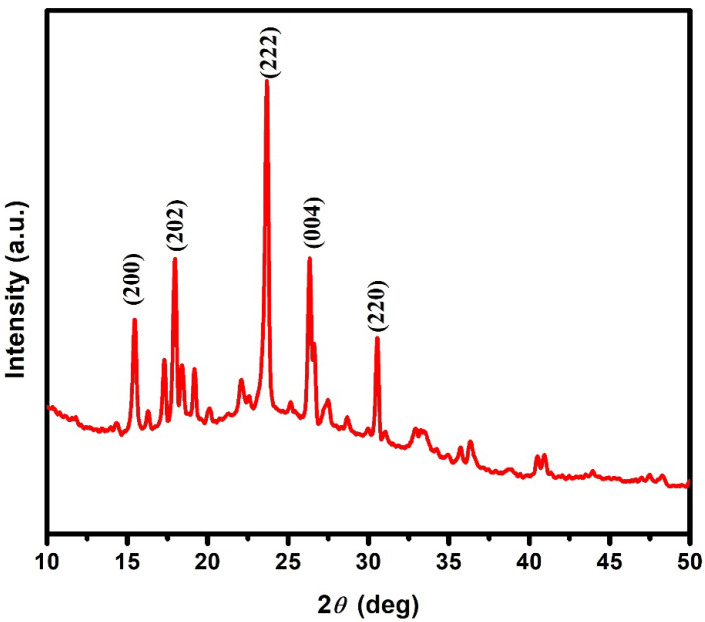
XRD pattern of as-synthesized PNA nanoparticles.

The as-prepared PNA NPs were evaluated according to their X-ray diffraction patterns, as represented in Figure 1. The major peaks at 15.5°, 18.2°, 23.2°, 26.3° and 31.4° corresponding to the hkl planes of (200), (202), (222), (004) and (220) observed in the XRD pattern indicated the crystalline structure of PNA [21]. The average crystallite size determined by the Scherrer equation [17] was found to be 14 nm.

#### 3.1.2. FTIR Studies

**Figure 2:**
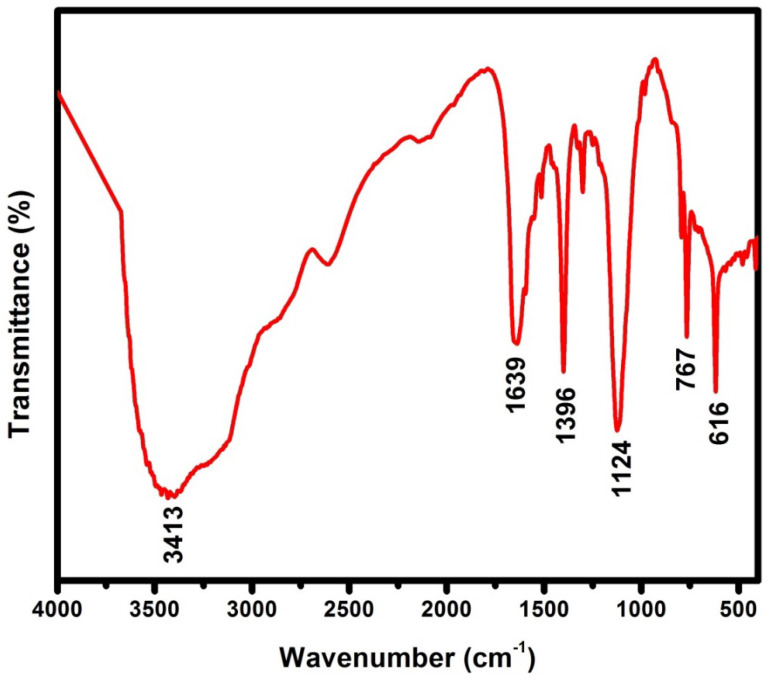
FTIR spectrum of as—synthesized PNA nanoparticles.

Figure 2 shows the FTIR spectrum of the as-prepared PNA. The peak observed at 3413 cm^−1^ was due to the hydrogen-bonded N-H stretching vibration, and another sharp peak at 767 cm^−1^ was noted for polymerization of 1-naphthylamine monomer to PNA [28,30,32]. The imine stretching was observed at 1639 cm^−1^ [31]. The peak at 1396 cm^−1^ was due to the benzoid vibration, and it was assigned to benzoid N-B-N and the quinonoid N=Q=N ring for PNA [29,32]. The peak at 1124 cm^−1^ noted for strong C-O stretching confirmed the presence of a secondary alcoholic group. The halogen compound C-Br stretching was observed at 616 cm^−1^.

#### 3.1.3. FESEM Analysis

**Figure 3:**
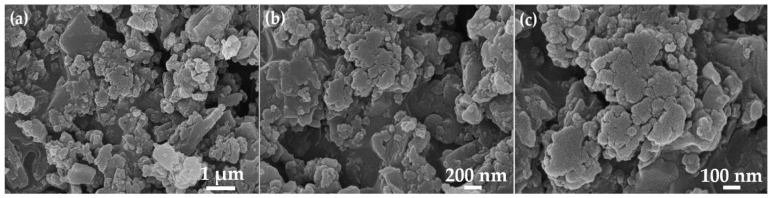
FESEM images of as—synthesized PNA nanoparticles at (a) 1 µm, (b) 200 nm and (c) 100 nm magnifications.

To determine shape and size, the synthesized PNA material was examined using field emission scanning electron microscopy (FESEM), and the results are presented in Figure 3. As observed from the FESEM images, nanoparticles with random sizes and shapes were observed in the PNA material; thus, one they can be referred to as PNA nanoparticles. Further, due to the highly dense growth, agglomerations of the PNA nanoparticles were seen. The shapes of the nanoparticles were random, and some spherical and elongated nanoparticles were observed in the micrographs.

#### 3.1.4. UV-Visible Analysis

**Figure 4:**
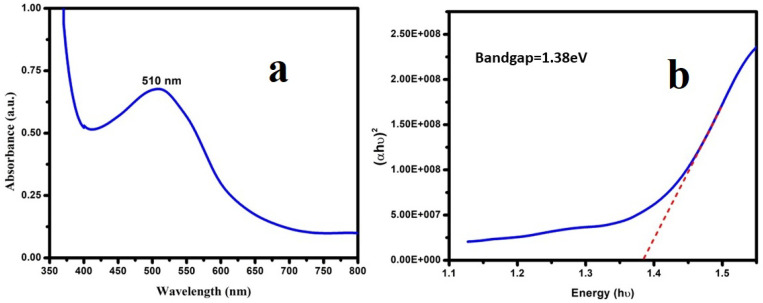
(**a**) UV-visible absorption spectrum and (**b**) bandgap energy of as-prepared PNA NPs.

Figure 4a shows the UV-visible spectra of as-prepared PNA nanoparticles. The observed UV–Vis spectrum exhibited wide absorbance peaks in the visible range centered at ~510 nm. The presence of a well-defined peak at 510 nm in the UV–Vis absorption spectrum was due the polaronic *n−π* * transitions [21,40,43]. Figure 4b shows a typical Tauc’s plot for the as-synthesized PNA nanoparticles. The calculated bandgap energy of the synthesized PNA nanoparticles was 1.38 eV, according to the Tauc’s plot [14].

### 3.2. Supercapacitor Application of PNA Nanoparticles

The electrochemical performances of the PNA-based electrode for supercapacitor were assessed using CV and GCD studies. The CV and GCD measurements were recorded with a potential window (Δ*V*) of −0.2 to −1.2 V at various scan rates and specific current values. The specific capacitance values of PNA were calculated from the *CV* using the following Equation (1) [44]:

(1) $$C_{sp}=\frac{\int idv}{s\Delta V.m}$$

where *C_sp_* is the specific capacitance (Fg^−1^), ∫idv is the integral area of the CV loop, *s* is the scan rate (Vs^−1^), Δ*V* is the potential window (V) and m is the mass of the electrode (g).

From GCD, the specific capacitance value was calculated using the following Equation (2) [44]:

(2) $$C_{sp}=\frac{I\Delta t}{m.\Delta V}$$

where *I* is the current (A), Δt is the discharging time (S), Δ*V* is the potential window (V) and m is the mass of the electrode (g).

The energy and power density were calculated using the following relation [8];

(3) $$E=\frac{1000\times C_{sp}V^{2}}{\left(2\times 3600\right)}(\mathrm{Wh}\;{\mathrm{kg}}^{-1})$$

(4) $$P=\frac{E\times 3600}{\Delta t}\;(W\;{\mathrm{kg}}^{-1})$$

where *E* is the energy density (Wh kg^−1^), *C_sp_* is the specific capacitance value obtained from either the CV or GCD (Fg^−1^), *V* is the potential window (V), *P* is the power density (W kg^−1^) and Δ*t* is the discharging time from the GCD curves (S).

**Figure 5:**
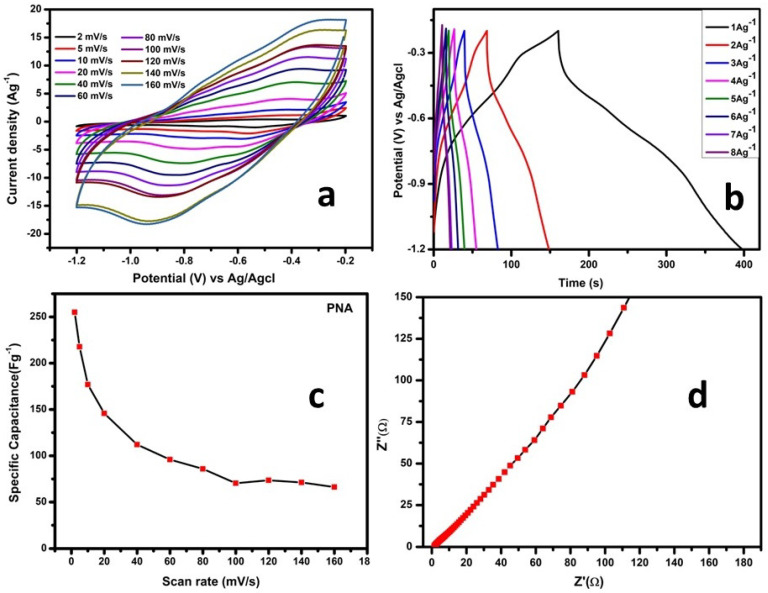
(**a**) Cyclic voltammetry, (**b**) galvanostatic charge–discharge, (**c**) scan rate vs. specific capacitance and (**d**) electrochemical impedance spectra for PNA nanoparticles.

Figure 5a shows the CV curve of PNA nanoparticles. The specific capacitance values calculated from the CV were 255, 217, 177, 145, 112, 96, 86, 73 and 66 Fg^−1^ at scan rates of 2, 5, 10, 20, 40, 60, 80, 120 and 160 mVs^−1^, respectively, as shown in Figure 5a. When the scan rate was increased, the redox peaks shifted noticeably. Even at a faster scan rate, broad redox peaks were found, confirming that a redox reaction was the major charge-storage event. The associated CV curves maintained their quasi-rectangular form as the scan rate was increased, confirming the electrode material’s high reversibility [9]. The maximum specific capacitance value calculated from the CV was 255 Fg^−1^ at a scan rate of 2 mVs^−1^. In order to study the charge/discharge behavior of the PNA electrode, GCD plots were produced, as shown in Figure 5b. The specific capacitance values of the PNA calculated from the GCD were 240, 162, 129, 112, 100, 96, 84 and 72 F g^−1^ at current densities of 1, 2, 3, 4, 5, 6, 7 and 8 Ag^−1^, respectively, as shown in Figure 5b. Figure 5c clearly shows that, by increasing the scan rate, the specific capacitance value slowly decreases. This might be because the mass transit of protons from the electrolyte to the electrode surface is kinetically limited, limiting the ion adsorption–desorption process at the electrode–electrolyte interface. The maximum energy and power density of pristine PNA were 33 Wh/kg and 500 W/kg. In Nyquist plots at higher frequency regions, the intersection point of the curve at the real impedance axis (Z′) determines the value of the effective series resistance (ESR). The ESR value of our PNA electrode was 1.5Ω, as shown in Figure 5d. From the EIS results, very small series resistance (Rs = 1.5 Ω) and a higher slope of the inclined line at the middle and lower frequency regions can be observed, which implies that our polymer sample possessed smaller diffusion resistance for electrolytic ions.

**Figure 6:**
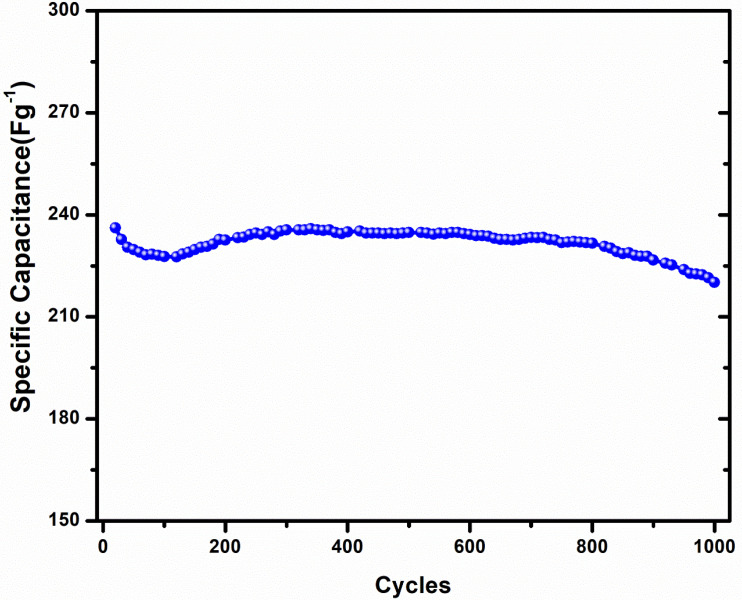
Cyclic performance of PNA nanoparticle electrode at a current density of 1 A g^−1^.

In order to analyze the cyclic stability of the PNA electrode, the cycling behavior of the PNA was studied over 1000 cycles, as shown in Figure 6. Long-term cycling of the PNA electrode resulted in a small decrease of ~7% in the specific capacitance, even after 1000 cycles. These cyclic stability results for the PNA-based electrode indicated that minor changes might occur in the physical or chemical structure during the charge–discharge cycling procedure.

### 3.3. Photocatalytic Properties of PNA Nanoparticles

**Figure 7:**
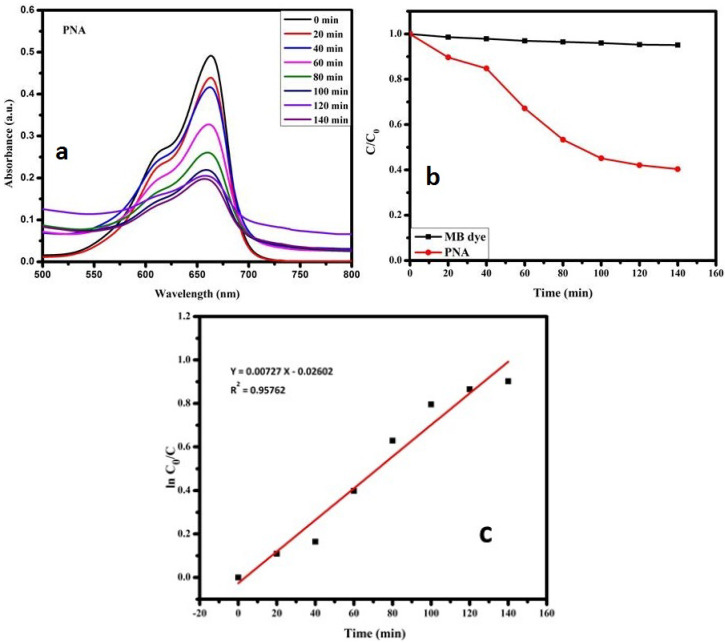
(**a**) Photocatalytic degradation and (**b**) degradation efficiency of PNA against MB dye; (**c**) first-order kinetics graph of MB dye photodegradation reaction.

The photocatalytic activity of PNA NPs was investigated for the degradation of methylene blue (MB) in the presence of UV irradiation at room temperature. A photocatalytic experiment was carried out using a UV source (λ = 376 nm, 11 W). The UV visible spectra of the MB dye showed a strong absorption peak around 665 nm (Figure 7a). The stock solution of the MB dye was prepared in a concentration of 5 mg L^−1^. Then, the PNA photocatalyst was added in the amount of 0.025 g for 100 mL of MB dye solution. The solution was left in the dark for 30 min to achieve adsorption equilibrium, and then the spectrophotometer was used to measure the absorbance readings. The UV light was then turned on, and the sample’s absorbance was measured every 30 min. The degradation efficiency was calculated using the following formula [2]:

(5) $$\%=\frac{C_{0}-C}{C_{0}}\times 100$$

where *C*_0_ is the initial concentration of the dye and *C* is the concentration of the dye after a time interval.

When UV light fell on the semiconducting PNA NPs, it induced the formation of electron–hole pairs (EHPs) through the photo-excitation of e^−^ from the valence band to the conduction band. The photo-generated e^−^ reacted with the dissolved molecular oxygen, forming a superoxide ion $O_{2}^{-}$. The superoxide ion $O_{2}^{-}$ further reacted with h^+^ to form a hydroperoxide radical $HO_{2}^{•}$.

$$\begin{array}{l} e^{-}+O_{2}\to O_{2}^{-}\\ O_{2}^{-}+H^{+}\to HO_{2}^{•} \end{array}$$

The photogenerated holes h^+^ react with $H_{2}O$ molecules and the hydroxide anions $\left(OH^{-}\right)$ to form highly active (•OH) hydroxyl radicals [40,45]:

$$h^{+}+OH^{-}\to •OH$$

$O_{2}^{-}$ and •OH have strong oxidation abilities, allowing them to completely oxidize the dye molecules into the final products $CO_{2}$ and $H_{2}O$.

Figure 7a,b show the graphs of the photocatalytic activity and degradation efficiency of PNA NPs against MB dye. Under UV light irradiation, MB dye was degraded by 61% within 140 min. The observed MB degradation percentage was much higher than that reported in the literature [46]. The high photocatalytic degradation of the synthesized PNA nanoparticles could have been mainly due to the small size of the nanoparticles, which possess higher surface area for larger dye adsorption. Furthermore, the small crystallite size of PNA NPs obtained in this work may have had a fundamental role in MB dye degradation. The electron–hole pair recombination played a vital role in the photocatalysis of the semiconducting PNA NPs. Rate kinetics is a critical parameter in degradation studies because it helps in estimating the rate at which a pollutant is removed from an aqueous solution. The rate constant can be calculated using the following equation [47]:

(6) $$\ln\frac{C_{0}}{C}=k_{app}t$$

A straight line with a slope equal to the first-order rate constant can be drawn by plotting ln (*C*_0_/*C*) versus the time interval, as shown in Figure 7c. For the as-prepared PNA NPs, the apparent rate constant (*k_app_*) and regression coefficient (R^2^) were 0.0073 min^−1^ and 0.9576, respectively. Similar rate constants were observed for degradation of red alizarin using PNA/ZnO under UV light [40].

## 4. Conclusions

This work demonstrated the chemical synthesis of pristine PNA from a NA monomer. XRD studies showed that PNA is crystalline in nature. FTIR confirmed the polymerization of 1-naphthylamine to PNA. The average particle size calculated from FESEM was found to be 22 nm. UV–Vis studies confirmed the transition molecular orbital ions of PNA. The results of the electrochemical studies showed that PNA possesses high specific capacitance values, as demonstrated by the CV curve, which was 255 Fg^−1^ at a scan rate of 2 mVs^−1^, and the GCD plot, which was 240 Fg^−1^ at a current density of 1 Ag^−1^. High energy and power density were obtained with the as-synthesized PNA NPs, and they showed a high cyclic efficiency of >93% after 1000 cycles. In addition, pristine PNA NPs exhibited remarkable degradation of MB dye under UV light irradiation, following the first-order rate kinetics.

## Data Availability

Not applicable.
